# Sample Size Calculation for Clinical Trials of Medical Decision Support Systems with Binary Outcome

**DOI:** 10.17691/stm2022.14.3.01

**Published:** 2022-05-28

**Authors:** O.Yu. Rebrova, A.V. Gusev

**Affiliations:** Professor, Department of Medical Cybernetics and Informatics; Pirogov Russian National Research Medical University, 1 Ostrovityanova St., Moscow, 117997, Russia; Professor, Department of Endocrinology, Institute for Higher Education and Additional Professional Training; Endocrinology Research Centre, 11 Dmitriya Ulyanova St., Moscow, 117292, Russia; Leading Researcher, Sector of Dynamic Neural Networks, Department of Neuroinformatics, Center for Optical-Neuron Technologies; Federal State Institution “Scientific Research Institute for System Analysis of the Russian Academy of Sciences”, 36/1 Nakhimovsky Prospect, Moscow, 117218, Russia; Chief Researcher, Laboratory of Evidence-Based Medicine and Biostatistics; Mental Health Research Center, 34 Kashirskoye Shosse, Moscow, 115522, Russia; Head of Business Development; K-SkAI LLC, 17 Naberezhnaya Varkausa, Petrozavodsk, The Republic of Karelia, 185031, Russia; Senior Researcher, Department of Scientific Fundamentals of Health Organization; Russian Research Institute of Health, 11 Dobrolyubova St., Moscow, 127254, Russia; Expert of the Sector of Clinical and Technical Trials; Research and Practical Clinical Center for Diagnostics and Telemedicine Technologies of the Moscow Health Care Department, 24/1 Petrovka St., Moscow, 127051, Russia

**Keywords:** clinical decision support systems, diagnostic models, predictive models, sample size, binary outcome, clinical trials, external validation

## Abstract

Currently, software products for use in medicine are actively developed. Among them, the dominant share belongs to clinical decision support systems (CDSS), which can be intelligent (based on mathematical models obtained by machine learning methods or other artificial intelligence technologies) or non-intelligent. For the state registration of CDSSs as software medical products, clinical trials are required, and the protocol of trial is developed jointly by the developer and an authorized medical organization. One of the mandatory components of the protocol is the calculation of the sample size.

This article discusses the calculation of the sample size for the most common case, the binary outcome in diagnostic/screening and predictive systems. For diagnostic/screening models, cases of a non-comparative study, comparative study with testing of the superiority hypothesis, comparative study with testing of a hypothesis of non-inferiority in cross-sectional studies are considered. For predictive models, cases of randomized controlled trials of the complex intervention “prediction + prediction-dependent patient management” with testing of the hypothesis of superiority and non-inferiority are considered.

It is emphasized that representativeness of the sample and other design components are no less important in clinical trials than sample size. They are even more important since systematic biases in clinical trials are primary, and even the most sophisticated statistical analysis cannot compensate for design defects. The reduction of clinical trials to external validation of models (i.e. evaluation of accuracy metrics on external data) seems completely unreasonable. It is recommended to perform clinical trials with the design adequate to the tasks, so that further clinical and economic analysis and comprehensive assessment of medical technologies are possible.

The sample size calculation methods described in the article can potentially be applied to a wider range of medical devices.

## Introduction

Currently, software products for use in medicine are actively developed. Among them, the dominant share belongs to clinical decision support systems (CDSS), which can be intelligent (based on mathematical models obtained by machine learning methods or other artificial intelligence technologies) or non-intelligent. According to the current legislation in Russia, such software is subject to state registration as medical device [1, 2], which, in turn, requires clinical trials. The purpose of these trials is to estimate the efficacy and safety of a medical device in terms of software [2], and they are performed in two forms:

1. research (analysis and evaluation of clinical data);
2. trials.

The meaning of term “research” in the document of the Ministry of Health of the Russian Federation dated August 30, 2021, No.885 [2] is not defined. According to the recommendations of the International Medical Device Regulators Forum [3], it is understood as “clinical assessment” as a combination of assessment of the reliability of clinical association, analytical validation, and clinical validation.

As a rule, the clinical trials program is created by the developer of the CDSS together with an external medical organization that has the right to perform such trials in accordance with current regulations.

Despite the fact that the goal of clinical trials is to evaluate the efficacy and safety of CDSS, in fact, an external validation of CDSS is currently performed instead, which allows assessing whether the performance metrics of the model declared by the manufacturer will be achieved on the data that were not used during training or testing of such a model.

From literature [4-6] and from our experience, it is known that when models are used in real clinical practice, degradation of their accuracy metrics (in particular, sensitivity — Se, specificity — Sp) is possible due to the fact that models begin to work in clinical cases unknown for them, which were not included to training and/or test datasets. This is due, among other things, to the fact that models are often trained on the data from only one medical institution, while the representativeness (typicality) of the sample used is often very doubtful, but the developers do not think about it. As a result, the generalizability of these models (the reliability of their work in other medical institutions) is quite low. Let us point out that external validation is very important, but it is absolutely insufficient for estimating efficacy and safety of CDSS.

When creating CDSS, models are designed mainly to solve diagnostic and predictive tasks (for example, to identify pathological lesions to MRI of various organs, to predict unfavorable events). At the stage of planning clinical trials and external validation, one of the difficult practical issues is to determine the size of the patient sample necessary and sufficient to elaborate reliable conclusions about the quality of the diagnostic or predictive model. Ultimately, this is necessary to build confidence in the results of clinical trials of a software product containing this model in the process of registering it as a medical device. Thus, correctness of determining the sample size can become the important factor in the success of clinical trials and the subsequent obtaining a marketing authorization from state agency Roszdravnadzor by the developer.

It is important to note that sample size is not the only fundamentally important characteristic of the sample: its representativeness is also important [7]. Representativeness of the sample can be successfully achieved using a probabilistic method of its formation (random selection, systematic selection, cluster selection, etc.), but in practice this approach is impossible in most cases. The following non-probabilistic sampling methods are commonly used: convenience sampling, sequential (continuous) sampling, volunteer sampling, quota sampling, etc. In this situation, it is important to pay attention to the prevalence (P, proportion, frequency) of the diagnosed (or predicted) condition in the dataset since the most important operational characteristics (accuracy metrics) of the model depend on it.

A sample should be formed from the same patient population in which the developed CDSS is supposed to be used — the target population. So, if a model is developed on the patient data from hospital case records, this product should be validated and used for the same patients and not for patients, for example, of an outpatients’ clinic. Ideally, the training sample should be also representative, at least in terms of the prevalence of the diagnosed (or predicted) condition. However, developers often deliberately achieve a balanced training sample (equality of the volumes of recognized classes) since in this case they get models with higher estimates of accuracy metrics. At the same time, developers often do not realize that failure will overtake them later, when the model is almost guaranteed to be inoperable in real practice, where the same class balance will not occur.

The description in publications and reports of the development and validation of diagnostic and prognosis models as a whole should comply with the modern recommendations STARD [8] and TRIPOD [9]. Both documents note that calculation of the sample size should be described. The literature discusses various ways to determine a sufficient sample size for the purposes of external validation of predictive and diagnostic models of artificial intelligence, depending on the outcome being studied (synonyms: outcome, function, dependent variable, output) [10-14]. Such outcomes may be a binary outcome, a categorical outcome (when three or more classes/events are recognized), a continuous outcome, or time to event.

In this paper, we consider approaches to calculating the sample size in clinical trials in order to assess the efficacy and safety of diagnostic and predictive models with binary outcome.

## Diagnostic models

The efficacy and safety of diagnostic models should be studied in a cross-sectional study. Its main features are as follows: diagnostic tests (at least new and reference) are applied to the patient simultaneously (with a minimum time interval), and their results are mutually blinded. The design of cross-sectional studies can be easily understood from the template for their description in STARD publications [8].

The choice of method for calculating the required sample size for binary outcome in a diagnostic/screening model depends on the answers to the following questions:

1. Is the study comparative or not?
2. If the study is comparative, which hypothesis is being tested?

Accordingly, depending on the answers to these questions, the following situations arise:

1. Binary outcome, non-comparative study — comparison with the reference test.
2. Binary outcome, comparative study:

- the hypothesis of the superiority of accuracy/safety of new test over routine test when compared to reference test;
- the hypothesis of non-inferiority/safety of new test in relation to the routine test when compared to the reference test.

Basically, in a comparative study, it is also possible to test the hypothesis of equivalence of new test and routine test when compared to the reference test. However, this situation is extremely rare, so we will not consider it here.

Before proceeding to the consideration of calculation methods, let us list the main metrics for assessing the quality of diagnostic models with a binary outcome in cross-sectional studies:

1. Sensitivity (recall in machine learning) and specificity are stable operational characteristics of a model, that are independent of the prevalence (frequency) of the identified condition in the target population. Their point estimates and also confidence intervals (CI) with 95%, and even better 99% confidence level should be calculated. Note that sensitivity and specificity vary reciprocally, and therefore, by optimizing one metric, we worsen the other one.
2. Positive and negative predictive values (PPV and NPV, respectively) are estimates that depend on prevalence of target condition, again their CI (95%, 99%) are also needed to be calculated. In machine learning, PPV is usually called precision. If the sample is representative to the target population in terms of prevalence (this is usually the case of consequtive or random sampling), the calculation of predictive values is simple. However, if positive and negative cases were sampled separately, an adjustment for prevalence is needed:

PPV=Se⋅P/[Se⋅P+(1−Sp)⋅(1−P)];NPV=Sp⋅(1−P)/[Sp⋅(1−P)+(1−Se)⋅P].

Predictive values are extremely important since the physician use them when evaluating the result of the diagnosis or prediction for a particular patient, given the probability of overdiagnosis and underdiagnosis.

In some cases, the overall accuracy of the model is also evaluated, i.e. the ratio of the sum of true positive and true negative results to the total number of observations in the sample. In machine learning, this metric is usually referred to as accuracy. Sometimes accuracy is also understood as the average between the Se and Sp values. For accuracy, CIs (95%, 99%) can also be calculated. Accuracy is also prevalence-dependent, so it cannot be calculated for a non-representative sample without correcting for prevalence. This metric is too general, not useful for doctors, so it is not recommended to use it.

Such a popular metric as the area under the receiver operating curve — AUROC — also has a general character. Let us emphasize that this metric is not binary and, accordingly, those calculations of the sample size, which will be discussed below, are not applicable to it. ROC analysis can be done both in [(1–Sp); Se] and in [(1–NPV); PPV] coordinates. The last analysis is preferable, as it is focused on the physician — the person who makes the decision regarding a particular patient. ROC analysis is often used for preliminary comparison of the accuracy of the models being studied, especially if there are many of them (which is often in case of building machine learning models). However, ROC analysis is completely insufficient to prove the efficacy of the model.

Next, a cut-off value should be defined if the model outcome has a continuous range. The criteria for this can be the following:

1. minimum type I error (overdiagnosis) with acceptable type II error (underdiagnosis);
2. minimum type II error (underdiagnosis) with acceptable type I error (overdiagnosis);
3. balance of Se and Sp;
4. maximization of their sum (Youden’s index), etc.

After determining the cut-off value, the calculation of Se, Sp, PPV, and NPV metrics for this selected value should follow. Usually, in diagnostic tasks, it is recommended to optimize Se and/or PPV and sacrifices (to an acceptable value) Sp and NPV. In screening tasks, it is vice versa: Sp and/or NPV are optimized with acceptable Se and PPV values. Note that the so-called one-sided use of the model (binary classifier) is possible, for example, the use of the model only to confirm the target condition (i.e., for diagnostics) if the PPV is high, the NPV is low, and at the same time the cost of type II errors (hypodiagnosis) is small. Conversely, a model with a high NPV and a low PPV can be used for screening if the cost of hyperdiagnostic errors is low.

### Binary outcome, non-comparative study of diagnostic/screening test

In the study of a new (index) test of diagnosis, the reference test should be the best test currently available for diagnosing the condition. It is assumed that the reference test ensures 100% diagnostic accuracy for all metrics. Histological examination is usually considered such a test in medicine, but it is invasive and in most cases cannot be used. However, reasons for the choice of the reference test should be given in reports and publications.

Diagnostic test metrics — Se, Sp, PPV, NPV — are proportions (fractions) for which it is necessary to estimate CI (usually, a confidence level of 95% is used). It is the lowest limit of CI that should be set as the parameter when calculating the sample size. Usually, one should strive to ensure that this limit is not lower than 85%. That is, the model is good if the CI for any of the metrics lies in the range of 85–100%. At the same time, it is obvious that if the CI includes 50% or even approaches this value, then the model is not useful, and instead of using it, it is easier to rely on chance by tossing a coin.

Thus, calculation of sample size in this case is reduced to solving the inverse problem — calculating the CI (usually 95%) for the expected metric value. With that, the target value of the metric should be set based on clinical significance, i.e. by physicians, not by statisticians. This means that it is physicians who should set the minimum acceptable value for the diagnostic accuracy index, with a hypothetical 100% accuracy of the reference test. Then, an acceptable value of the alternative metric should also be set (NPV is the alternative metric for PPV, PPV is the alternative metric for NPV). The sample sizes obtained for the two alternative metrics should be summed up.

Higher requirements should be imposed on PPV (given the prevalence) if the problem of diagnostics is being solved, i.e. identifying a high-prevalence condition in the target population. If the task of screening is being solved, i.e. identifying a low-prevalence condition in the target population, one should primarily focus on NPV. The Se and Sp are less important from the practical point of view of the use of the CDSS, while the Se metric is associated with the PPV, and Sp is associated with the NPV.

Manual calculating of CI is complicated, so we do not give the formula here. Of course, any professional software package has convenient options for such calculations. However, you can use the not very convenient but reliable online calculator https://www.graphpad.com/quickcalcs/confInterval1/ (although, there are many other similar calculators) using the procedure\ of “mathematical adjustment” of the numerator and denominator values for a given proportion.

#### Example 1.

Physicians set acceptable values 90% for PPV and 80% for NPV. This means that the lowest limit of CI for PPV should be at least 90%, for NPV — at least 80%. Then one can approximately assume that the point estimate of PPV is located in the middle of the interval between 90 and 100%, i.e. is equal to 95%. Note that for small samples, this assumption is not justified. Provided that the future sample will be representative at least in terms of the prevalence of the target condition, the required sample size obtained using the above calculator will be 150 patients since the 95% CI for PPV calculated by the exact Clopper–Pearson method in this case is equal to (90.6%; 98.1%). The same applies to NPV: the middle of the interval between 80 and 100%, i.e. 90% can be taken as a point estimate of the metric. The sample size for NPV calculated using the same calculator would be 63 — for obtaining a 90% proportion with a 95% CI (80.5%; 95.9%). After summing 150+63, we get 213 as the final value.

Next, the obtained sample size should be distributed between positive and negative cases (determined by the reference test) in accordance with the prevalence of the condition in the target population.

#### ***Example 2*** (continuation of Example 1).

If the prevalence of the condition in the target population is 60% (0.6), then 213·0.6=128 patients should be included in the case group, 213–128=85 patients in the comparison group. If the prevalence of the condition in the target population is 10% (0.1), then the distribution will be different: 213·0.1=21 patients in the case group, 213–21=192 patients in the control group.

It should be emphasized that if the model was developed on the so-called balanced training set, i.e. this sample was not representative in terms of prevalence, then the estimates of PPV and NPV obtained during internal testing are biased — and the more the actual prevalence deviates from the group sizes in the training sample. As a result, it will be difficult, if not impossible, to obtain the same values in a well-designed sample during clinical trials. With that, Se and Sp do not depend on the prevalence, and therefore they are easier to reproduce, but they have no practical value for doctors.

External validation of the diagnostic/screening model, which often, unfortunately, replaces clinical trials of such models, actually corresponds to the non-comparative study design described above: samples of positive and negative cases are formed, and Se and Sp are calculated. This can be considered acceptable if the following principles are keeped:

1. sample is obtained strictly from target population;
2. reliable reference test is used;
3. the ratio of positive and negative cases correspond to the prevalence of the condition in the target population;
4. calculation of not only Se and Sp but also of PPV and NPV is performed;
5. 95% CI must be calculated for all metrics;
6. safety is assessed, first of all, the consequences of under- and overdiagnosis errors.

### Binary outcome, comparative study, hypothesis of superior accuracy/safety of diagnostic/screening test

The hypothesis of the superiority of the model over the existing test is tested when compared to the reference test. Thus, diagnostics is performed by three tests — reference, new, and old (routinely used, suggested for replacement) ones.

In this case, the sample size calculation is based on clinically significant superiority of the new test over the old one. The main calculation parameters are:

1. type I error (alpha) — usually set to 5%;
2. statistical power — 90% is recommended, 80% is minimum;
3. the value of the chosen metric (for example, PPV) for the old test;
4. the value of the chosen metric for the new test.

It should be noted that the accuracy of the routinely used test is not always known. In this case, one should perform a preliminary study with assessment of its accuracy.

If the excess of the new test accuracy over the old one is expected to be small (for example, 5%), such a test is unlikely to be introduced into medical practice. A new test is often more expensive than the routinely used one; accordingly, in this situation, a clinical trial should be followed by a clinical and economic analysis, during which the incremental cost effectiveness ratio is to be evaluated. In other words, it must be determined whether the cost increment is justified in terms of the increment in diagnostic accuracy. Besides, medical practice is generally very conservative, and a small improvement in accuracy may not be a strong argument in favor of introducing a new test. So, to calculate the sample, it is necessary for physicians to establish the minimum accuracy value (compared to the routine test) that would convince them to use a new, potentially more accurate test.

Calculation of the sample size is possible in various statistical packages, but reliable online calculators, for example, https://sealedenvelope.com/power/binary-superiority/ (of course, with reference to the calculator and the literature that underlies the calculations and is listed on the calculator webpage) can also be used.

#### Example 3.

A new diagnostic test has been developed, which exceeds the old one in terms of accuracy by 10%. The accuracy of the old test (in the control group interface) is 80%, of the new one — 90%. Then the required sample size (with a type I error of 5% and a statistical power of 90%) is 263 patients.

### Binary outcome, comparative study, non-inferiority of accuracy/safety of diagnostic/screening test

The hypothesis is tested that the accuracy of the new test is not lower than the accuracy of the old test. Of course, the question may arise, why then a new test is needed at all. However, for any medical technology, not only efficiency is important (accuracy in the case of diagnostics or screening), but also safety. Then the increase in safety can also be proven in clinical trials — when testing the hypothesis of superiority in relation to the criterion (or several criteria) of safety. This is especially important if the old test is invasive or if it requires radiation of the patient. In addition, the economic aspect is also important. Thus, a new test may be cheaper with the same accuracy, which will create an argument in favor of implementing this new test.

In this case, diagnostics is also done using three tests — reference, new, and old (routinely used, proposed for replacement). The sample size calculation is based on clinically significant non-inferiority of the new test to the old one. The main calculation parameters are:

1. type I error (alpha) — usually set to 5%;
2. statistical power — 90% is recommended, 80% is minimum;
3. the value of the chosen assessment metric (for example, PPV) for the old test;
4. the value of the chosen assessment metric for the new test;
5. the threshold of non-inferiority.

The last parameter shows the difference between the values of the estimated metric, which can be considered acceptable by physicians. For example, if the new test must be exactly the same as the old one (the accuracy of the old and new tests is 80%), the threshold is zero. Proving this would require an infinite number of observations. By increasing the threshold, we admit that the new test may still be somewhat worse than the old one. The larger this difference, the easier it is to prove non-inferiority since the required sample size will decrease.

Sample size calculation is possible in the online calculator on the same online service (https://sealedenvelope.com/power/binary-noninferior/).

#### Example 4.

The accuracy of the new and old tests is set at 80%, the threshold is 5% with type I error of 5% and statistical power of 90%. Then 1097 patients would be required to prove this hypothesis. If the threshold is set to 7%, the required number of patients in the sample will be almost half smaller — 560.

It is possible that the new test is slightly better than the old one by a clinically significant amount (e.g., by 2%). Then the required sample size will be smaller.

#### Example 5.

The accuracy of the new test is 82%, of the old one — 80%, the threshold is 5%, the type I error is 5%, and the statistical power is 90%. Then 538 patients will be required.

Note that sample size when testing the hypothesis of non-inferiority is always larger than when testing the hypothesis of superiority.

## Prediction models

Such models are much more difficult to assess. First of all, one should determine how the prediction will be used. It is typically used to change the patient management for secondary or tertiary prevention. Thus, testing such a model actually is testing of combined medical technology “prediction + prediction-dependent patient management”. Moreover, if the prediction is accurate, but there are no effective and safe ways to prevent outcomes (for example, to prevent unfavorable events), then there is no background for a prediction. Moreover, a negative prediction will be harmful if the patient is informed about it.

Let us note once again that it is important to have an effective intervention of influencing on outcomes exactly at the stage of the disease/life at which the prediction is made. It is known that treatments that are effective in the later stages of the disease may be completely useless in the early stages of the same disease. Thus, starting to solve the problem of prediction, one must first make sure that there are effective ways to prevent predicted unfavorable events.

Another important parameter of predictive models is term for prediction, which is specific for a particular task. Sure, we are talking about the prediction period not exactly for 1 year, 5 years, etc., but a period of up to 1 year, up to 5 years, etc. The shorter the prediction period, the easier it is to build it — this is due to the completeness of the data, the absence of historical bias, etc. For example, predicting the outcome of hospitalization due to an acute illness is much easier than predicting myocardial infarction in up to 5 years.

Predictive models should be evaluated using another study design — a randomized controlled trial (rather than cross-sectional design for diagnostic/screening tests).

The main features of such trials are the following:

1. the target population is synchronized by some event (diagnosis, certain age, surgical intervention, etc.);
2. patients after signing the informed consent are randomized into the main and control groups;
3. in the main group, prediction is done for all patients, and in case of an predicted bad outcome, the patient is managed using modified strategy compared to the routine one (for example, more frequent visits to the doctor for early detection of recurrence after surgery); with a predicted good outcome, routine or even simplified management is used;
4. in the control group, prediction is not performed, the management tactics are routine;
5. an observation period is established, during which adverse events are recorded. The duration of observation should be such that a sufficient number of predicted outcome in the control group occurs.

The performance metrics of the model in such trials are relative risk and absolute risk reduction. If there was a large dropout from the study (and this is an inevitable companion of long-term observation, which is necessary for slowly accumulating events), then it is required to evaluate another metric — the hazard ratio (we do not dwell on this case in this article). The tested hypotheses are superiority or non-inferiority. Let us consider the above situations one by one.

### Binary outcome, prediction model, superiority hypothesis

In developing predictive models, it is usually assumed that if a prediction is available, it will be possible to improve the patient outcomes. Usually, bad outcome is predicted in order to reduce its frequency in the main group compared to the control group through the use of some medical prevention technology — secondary (prevention of the disease) or tertiary (prevention of complications, relapses, exacerbations, progression, disabling, etc.).

In this case, sample size is calculated the same way as described above for the superiority hypothesis, however, two samples are needed here, each of which will consist of the calculated number of patients. Calculations can be done in the calculator at https://sealedenvelope.com/power/binary-superiority/. It is also possible to form samples of unequal size (for example, in a ratio of 3:1), but the statistical power then decreases and, therefore, a larger sample size is required.

#### Example 6.

In the control group, the disease occurs in 20% of the patients, in the main group, we would like it to occur in no more than 10% of the patients (the latter value is chosen in accordance with the expectations of doctors, i.e. clinical significance of the effect). Then, in case of type I error of 5% and statistical power of 90%, the success rate (no disease) in the control group of 80% and the success rate in the main group of 90%, the required size of each of the groups will be 263 patients.

### Binary outcome, prediction model, non-inferiority hypothesis

In such trials, it is usually assumed that if there is a prediction, it will be possible to simplify the patient management without worsening the outcomes (development of the disease, complications, etc.). For example, it is possible to invite a patient to a visit not once a year after surgery, but once every 2 years, without worsening the outcome.

Sample size is calculated the same way as described above for the hypothesis of non-inferiority, but now two samples are required, and each sample should include the calculated number of patients. Calculations can be done in the calculator at https://sealedenvelope.com/power/binary-noninferior/.

#### Example 7.

In the control and experimental groups, the disease occurs in 20% of the patients (i.e. the rate of “success” in both groups is 80%), while the threshold of non-inferiority is set at 5%. Then, with type I error of 5% and statistical power of 90%, the required number of patients in each group is 1,097.

External validation of the predictive model, to which clinical trials of predictive models are now unreasonably reduced, is done in the design of a retrospective case–control study: the main and control samples are formed according to the presence/absence of predicted outcome (event), patient data are extracted for a period corresponding to the prediction period, and the accuracy of the prediction (in terms of AUROC, Se, Sp) is evaluated. This approach is fraught with serious biases that do not allow to correctly assess the efficacy and safety of the model, in particular:

1. the cohort is not synchronized;
2. patients with missed data (both in the data used for prognosis and in outcomes) are not included in the analysis;
3. the prediction period is fixed, while events in patients occur at different times;
4. all medical interventions that were applied to patients during this period are ignored.

Thus, the design of a retrospective case–control study is completely unsuitable for assessing a predictive model. A palliative measure could be a retrospective cohort study, the main design features of which are as follows:

1. the target population and cohort synchronization criterion are specified;
2. based on data at the sync point, a prediction is built, positive cases are included in the main group, negative cases — in the control group;
3. the frequencies of the outcome in the groups that have arisen over the prediction period, and predictions are compared; dropouts and medical interventions are taken into account.

The predictive quality metrics in this situation are Se, Sp, PPV, and NPV, and the calculation of the sample size is reduced to the case of incomparable study of the diagnostic/screening test. During prediction, overdiagnosis is often preferred as a conservative tactic.

Of course, a retrospective cohort study is only the lesser of the two evils since it does not provide unbiased estimates of the efficacy and safety of the predictive model either.

## Conclusion

We have considered sample size calculation for the most common type of models — the one with a binary outcome. However, in any calculation, it is still desirable to increase the number of patients by 5–10% for reliability, especially if dropouts are possible, or the statistical power is set at 80%.

Sample size calculation is only one of the components of clinical trials protocol, which are planned jointly by developer and authorized medical organization. Other aspects of clinical trial design are equally or even more important since systematic biases in clinical trials are primary and even the most sophisticated statistical analysis cannot compensate for design defects. Clinical trial reduction to external validation of models seems completely unreasonable. It is recommended to perform clinical trials with adequate design, so that further clinical and economic analysis, and comprehensive assessment of medical technologies are possible.
